# B cells as a target of immune modulation

**DOI:** 10.4103/0972-2327.58275

**Published:** 2009

**Authors:** Kathleen Hawker

**Affiliations:** The Ohio State University Medical Center, 2050 Kenny Road, Suite 2250, Columbus, Ohio 43221, USA

**Keywords:** B cells, multiple sclerosis, treatment

## Abstract

B cells have recently been identified as an integral component of the immune system; they play a part in autoimmunity through antigen presentation, antibody secretion, and complement activation. Animal models of multiple sclerosis (MS) suggest that myelin destruction is partly mediated through B cell activation (and plasmablasts). MS patients with evidence of B cell involvement, as compared to those without, tend to have a worse prognosis. Finally, the significant decrease in new gadolinium-enhancing lesions, new T2 lesions, and relapses in MS patients treated with rituximab (a monoclonal antibody against CD20 on B cells) leads us to the conclusion that B cells play an important role in MS and that immune modulation of these cells may ameliorate the disease. This article will explore the role of B cells in MS and the rationale for the development of B cell–targeted therapeutics. MS is an immune-mediated disease that affects over 2 million people worldwide and is the number one cause of disability in young patients. Most therapeutic targets have focused on T cells; however, recently, the focus has shifted to the role of B cells in the pathogenesis of MS and the potential of B cells as a therapeutic target.

## Introduction

B cells go through several stages of development, characterized by both an antigen-dependent and an antigen-independent phase. B cells arise from stem cells in the bone marrow. After a series of processes, pro-B cells are initially formed; these then develop into pre-B cells and, finally, into immature B cells, all of which are antibody independent and can be characterized by specific surface markers and adhesion molecules. Immature B cells can express IgM and migrate to the peripheral lymphoid tissue where they mature into naïve B cells, expressing IgM and IgD. Through clonal expansion and a maturation process, the cells are able to produce antigen-specific antibodies. Activation of the cells occurs after exposure to a specific antigen and to co-stimulation molecules, which promotes migration to the germinal centers in the lymph nodes and differentiation into memory B cells, the shorter-lived plasmablasts, and the longer-lived plasma cells.[1,2] Negative selection occurs at various stages throughout these processes in order to promote self-tolerance. Loss of this regulation can result in autoreactive cells, which would lead to production of antibodies to self-antigens.[3]

## B cells and autoimmunity

B cells are involved in autoimmunity through a variety of mechanisms. B cells can present antigen to T cells and, in conjunction with co-stimulatory molecules, may cause activation of naïve T cells and, ultimately, secretion of proinflammatory cytokines and chemokines. Because B cells can specifically bind and concentrate antigen to a much greater degree than other antigen-presenting cells, they are more efficient at presenting antigen to T cells, thus can prime potentially autoreactive cells. Once T cells are activated, B cells may themselves be activated by interactions between the T cell receptor and ligands on the B cells. The activated B cells may increase production of Th17 cells and T cell–secreted cytokines may increase antigen-specific antibody formation by B cells. B cells morph into plasma cells, which secrete antibody and may bind complement, and both may contribute to tissue destruction.[1,4]

B cells may also be activated by a T cell–independent mechanism. Toll-like receptors or polysaccharides may induce antigen-specific responses and result in a different population of B cells (B2 cells).[5]

## Animal models

The animal model of MS – experimental allergic encephalomyelitis (EAE) – can be induced by myelin oligodendrocyte protein (MOG), a myelin protein found on the outer surface layer of the lamella, through either passive transfer or active immunization. In mice and marmoset animal models, MOG induced a strong B cell response, and immunolabelling revealed evidence of B cells and plasma cells specific for MOG in lesions; in addition, antibodies specific to MOG were found to bind to disintegrating myelin.[6–8] This data would suggest that antibody formation, after a B cell response, was integrally involved in myelin destruction.

There is conflicting evidence regarding the role of B cells in EAE. EAE could be induced by MOG peptide in B cell–deficient mice and the degree of inflammation and severity of disease was similar in both the deficient and wild-type mice. On the other hand, Lyons *et al*. used an MOG protein complex to induce EAE in both B cell– and plasma cell–deficient mice. EAE occurred when the protein complex was used and, although intense deposition of macrophages and memory and activated T cells was seen, no active demyelination was demonstrated. This finding corroborated the above evidence that B cells play an important role in tissue destruction in lesions but are not necessarily needed for EAE induction.[9–12]

## Immunology

There are several lines of evidence to prove that B cells are involved in the pathogenesis of multiple sclerosis (MS). It has long been known that the intrathecal synthesis of immunoglobulin, without a corresponding increase in peripheral blood, occurs in greater than 90% of MS patients.[13] There is a skewed kappa/lambda ratio in the cerebrospinal fluid (CSF) of MS patients and flow cytometry of CSF revealed a marked predominance of memory B cells in contrast to the usual ratio of naïve to memory B cells seen in peripheral blood. Electrophoresis may identify oligoclonal bands (IgM and IgG) in the CSF and several studies have suggested that patients with these bands in their CSF have a worse prognosis than those patients who are oligoclonal band negative.[15–24] In one study, patients with clinically definite MS and positive oligoclonal bands had significantly higher expanded disability scores than those who were oligoclonal band negative. Another study examined the B cell to monocytes ratio in the CSF and found a more rapid progression of disability in those patients with a higher ratio.[25–27] In a study by Cepok *et al*. there was correlation between plasmablasts (which are present in large numbers in the CSF) and disease activity as demonstrated on magnetic resonance imaging (MRI).[28]

There is evidence that B cells in the CSF of MS patients have a greater degree of CD80, a co-stimulatory molecule;[29] also, B cell activating factor (BAFF), a cytokine essentially for B cell survival, is upregulated in the CSF of MS patients as compared to CSF of controls.[30] Other B cell chemokines, such as CXCL12, CXCL13, and α-lymphotoxin, have been found in MS lesions.[31]

Pathological studies from autopsy and biopsy material in MS patients have revealed the presence of B cells, plasma cells, and antibodies in active lesions. Lucchinetti described four types of patterns, and the most common pattern seen among all subtypes (pattern II) showed intense deposition of plasma cells, antibody, complement, and macrophages.[32]

Serafini *et al*. have documented evidence of ectopic germinal-like centers at autopsy in secondary progressive MS patients and more recent studies have documented the same findings in biopsies from RRMS and PPMS patients. These germinal-like centers were composed mainly of B cells and evidence of actively replicating Epstein-Barr virus (EBV) virus was documented.[33,34] Previous studies have shown that B cells can act as a chronic repository of EBV, as the virus binds to a receptor on the B cell surface.[35] Although it is unclear what role the germinal-like centers play, it is postulated that EBV may chronically stimulate the immune system within the CNS.

## Evidence for B cells as a therapeutic target

There are numerous lines of evidence that B cells are affected by treatments in MS. Downregulation of B cell MHC class II and a diminished antigen-presenting capacity (APC) was demonstrated after the use of high-dose steroids (methylprednisolone). Intravenous gamma globulin inhibits B cell differentiation and cytokine release as well as antibody production. Although the ligands are not specific to B cells, interferon-β downregulates CD80 and CD40 expression. Both glatiramer and interferons can reduce APC capacity, and natalizumab blocks VLA-4 on B cells, preventing transport into the CSF. Mitoxantrone, and to a lesser degree cyclophosphamide, causes B cell death.[30,36–42] Despite these multiple effects on B cells, most of these medications were thought to have their primary effect on T cells and it has been unclear whether the other effects on B cells played a role in the ultimate outcome of drugs on the disease.

The pivotal role that B cells played in the pathogenesis of MS was not clear until the results of the phase II, double-blind, randomized, placebo-controlled trial of rituximab in 104 patients (*n* = 69 on rituximab and *n* = 35 on placebo) with relapsing–remitting MS (RRMS) was released. Rituximab is a chimeric monoclonal antibody that targets CD20, a specific ligand on B cells only. CD20 is expressed from the pre B cell to memory B cells; it is mainly lost at the plasmablast stage and is not expressed on plasma cells. It causes an almost complete depletion of peripherally circulating B cells through the mechanisms of antibody-dependent cell-mediated cytotoxicity (ADCC), complement-dependent cytotoxicity (CDC), and apoptosis.

In the phase II trial, patients received 1 gm of intravenous rituximab or placebo followed, 2 weeks later, by another 1 gm of drug or placebo. All patients were followed for 48 weeks. B cells were rapidly depleted within 2 weeks. Brain MRI was done at baseline and at weeks 12, 16, 20, and 24 and showed a profound effect on new gadolinium-enhancing lesions on MRI, with a decrease of 91% (*P* < 0.0001) as compared to placebo. The proportion of patient relapsing was reduced by 58% (*P* = 0.02) as compared to placebo; this effect persisted for up to 9 months and returned close to baseline by 11 months, despite the fact only one course of rituximab was used. This observation was replicated in a smaller open-label trial of rituximab in RRMS, where patients received two treatment doses: the first at baseline and the second at 6 months. New gadolinium-enhancing lesions were markedly suppressed throughout the follow-up period of 72 weeks and the relapse rate was also decreased from the baseline of 1.27 relapses per year to 0.12 relapses at weeks 24 and 48, rising slightly by week 72 to a rate of 0.23.[44] The safety profile, despite profound depletion of B cells, was good; though there was an increase in number of infusion reactions in the treated group, the difference between the two groups was not significant and the drug had little effect on immunoglobulin levels.[43]

A previous small open-label trial of rituximab done by Cross *et al*. in RRMS patients who were failing glatiramer or interferon, demonstrated that the peripheral depletion in B cells decreased not only CSF memory B cells but also T cells within the CSF.[45]

Although it was initially postulated that rituximab would have an effect in MS by gradually affecting antibody levels, the rapid effect seen in the phase I and II trials suggests that the primary action of the drug was through the B cells' role as antigen-presenting cells. Decreased antigen presentation would produce a lesser degree of T cell activation, decreased cytokine production, and reduced trafficking of B and T cells into the CSF and CNS. This theory would explain the observation of reduced numbers of T cells within the CSF after treatment with rituximab as seen in Cross's trial.

A more recent phase I/II randomized placebo-controlled double-blind trial of rituximab was done in 439 primary progressive MS (PPMS) patients. Subjects were randomized to a 2:1 treatment protocol, where patients received four courses of rituximab or placebo over a 2-year period, with a total follow-up of 122 weeks. Although there was no difference in the disability progression, as measured by the expanded disability scale, between the placebo and treated groups, subgroup analysis demonstrated that rituximab could significantly slow disability progression in patients 51 years old or less, in patients with a gadolinium-enhancing lesion on brain MRI, and in patients aged 55 years who had a gadolinium-enhancing lesion on brain MRI. Overall, there was a very significant reduction in mean change in T2 lesion volume on the MRI in treated *vs* placebo patients (*P* < 0.0008).[46]

There was no difference in incidence of nonserious infections between the two groups and only a very mild increase in serious infections in the rituximab-treated group. Infusion reactions were higher in the treated group but, by the time of the second course of treatment, this had fallen to the level seen in the placebo group. There was a mild decrease in IgM levels (31% *vs* 6% in placebo) seen at any time point in the trial, though decreases in IgG levels and IgA levels were comparable in the rituximab-treated and placebo-treated patients. The decreased immunoglobulin levels did not predispose patients to infection.[46]

The positive effect of slowing of disability progression seen in patients with evidence of inflammation on their MRI scans seem to imply that B cells have a role as antigen presenting cells in the progressive forms of the disease as well and that progression may be driven not only be neurodegeneration but also by inflammation, albeit to a lesser degree than seen in RRMS. It is not clear whether B cells have an independent role in the pathogenesis of progressive disease or whether the effect seen in the trial was mediated through alteration of autoreactive T cells.

Other B cell targeted agents are in early-phase trials to assess efficacy in RRMS.

Atacicept [transmembrane activator and calcitonin-modulating cyclophilin ligand receptor (TACI) immunoglobulin] targets the B cell survival factor BAFF as well as APRIL, a B cell proliferation-inducing factor. After treatment with atacicept, B cell development is arrested at the immature transitional T1 phase in the spleen. This produces fewer transitional T2 marginal zone and mature B cells in the spleen and immunoglobulin levels are lower. A trial of atacicept is underway and the results are awaited.

Ocrelizumab is a fully humanized anti-CD20 monoclonal antibody that works through a mechanism similar to that of rituximab; it is currently in a phase II trial to assess efficacy in RRMS in decreasing new gadolinium-enhancing lesions. Development of other anti-CD20 molecules is underway as well.[2,47–49]

Other B cell–depleting agents may have potential to affect disease in MS. Epratuzumab is a humanized monoclonal antibody that targets CD22 on B cells. Peripheral depletion of B cells is less complete than is seen with anti-CD20 therapies and studies suggest that epratuzumab may work mainly through immunomodulation of B cells rather than ADCC.[50–52]

DC2219 is a recombinant immunotoxin that binds bi-specifically to CD19 and CD22 and causes cytolysis of the B cell. Trials are currently underway to assess efficacy in lymphoma; however, the drug may have potential in MS as well.[53]

Belimumab is a fully humanized monoclonal antibody that targets BAFF. BAFF, a survival factor for B cells, is elevated in the CSF of MS patients (and many other autoimmune diseases) and treatment with belimumab decreases B cell numbers. Some efficacy against lupus has been documented with the drug and its alternative mechanism of action for B cell depletion may prove useful in MS.[54]

Abatacept is a soluble fusion protein, linking the Fc portion of IgG1 with the extracellular domain of human CTLA4. CTLA4 acts as a co-stimulatory molecule, binding the same molecules as CD28, and binding of the CTLA4 sends a negative signal for T cell activation. Abatacept also blocks co-stimulatory interactions between B and T cells by blocking B7. An early-phase trial on 127 RRMS patients using a dose of 2 mg/kg and 10 mg/kg was terminated early when increases in gadolinium-enhancing lesions and relapses were noted in the 2 mg/kg group. However, the 10 mg/kg group showed a decrease in MRI lesions and exacerbations as compared to placebo, and the drug still holds promise as a therapeutic agent in MS.[55–61]

Overall, the safety profile of these drugs has been impressive, with infusion reactions being the most common side effect; these reactions are seen initially and decline once ongoing B cell depletion is achieved. Small increases in infection rates and variable drops in immunoglobulin levels, which depends on the drug used, have been noted.[43] However, it is not known whether the safety profile will change with persistent long-term depletion of B cells. One may postulate that plasma cells populations, and thus immunoglobulin levels, may ultimately decrease, potentially leading to an increased risk of infection and tumor genesis.

In conclusion, it seems that B cells play many roles in perpetuating MS, e.g., antigen presentation, cytokine secretion, antibody production, and as a harbinger of EBV infection. At present, it is not clear which population of B cells (lymphoid *vs* peripheral, CSF memory or peripheral memory B cells) is the main contributor to the pathogenesis of the disease and further research is needed to further define the role of the B cell. It is clear, though, that B cell therapy holds great promise in the treatment of MS.
